# Reversed Palmaris Longus: A Rare Anatomical Phenomenon Discovered

**DOI:** 10.7759/cureus.38424

**Published:** 2023-05-02

**Authors:** Ayman Behiery, Mohamad Bakir, Ahmad Dawalibi, Asem H Elhossiny

**Affiliations:** 1 Department of Anatomy, College of Medicine, Alfaisal University, Riyadh, SAU; 2 College of Medicine, Alfaisal University, Riyadh, SAU

**Keywords:** muscle variant, muscle anomaly, inverted, reserved, forearm flexor, anatomical variations, palmaris longus

## Abstract

The palmaris longus (PL) muscle is considered by many to be a vestigial muscle due to it having little to no functional significance on the upper limb. This, however, made it highly valuable in surgical procedures, especially as a graft in plastic and reconstructive cases. Variations in the muscle’s morphology were discussed in the literature, but some are more rare than others. Those variations may have clinical implications on different pathologies such as Guyon’s syndrome or Carpal tunnel syndrome based on the nerves and vessels surrounding it, and thus demand a proper understanding of the variation’s anatomy. Here, we report a case of one of the rarer variations, a unilaterally reversed palmaris longus muscle in the left forearm of a 55-year-old male cadaver, discovered in a routine teaching session. Throughout the case, we will discuss the normal anatomy, the variation, and the clinical implications this variation may have.

## Introduction

The palmaris longus is a small muscle that has a spindle-shaped belly and can be found on the anterior forearm. It originates via the common forearm flexor tendon from the medial epicondyle of the humerus and is located between the flexor carpi radialis and the flexor carpi ulnaris, with the palmar aponeurosis being the most common insertion site. Even though the structure of the palmaris longus varies widely, it can generally be divided into three parts: a proximal part made up of tendon, a middle part made up of muscle that is spindle-shaped, and a distal part made up of a slender long tendon [1, 2]. The palmaris longus muscle has limited utility and functional significance, yet is commonly harvested for tendon reconstruction surgery because of its properties, one being how superficial it is in relation to the forearm, and another being the thin distal tendinous part it has. This makes it perfect to harvest in many surgeries [1, 2].

The palmaris longus muscle is primarily supplied by branches from the ulnar artery, and less commonly, through branches from the main brachial artery itself [3]. Embryologically, myotome complexes and dermatomes are developed from the condensation of mesenchyme, after which the myoblast develops from the migrated myotomes that went into the developing limb buds. The limb buds get elongated, and the muscle forms from the myoblasts. These two processes shape the muscle into their respective muscle groups through compartmentalization, an example of this being the palmaris longus muscle under the forearm wrist flexor group [4]. While generally observed in most individuals, this muscle can be missing in a certain proportion of people. Its existence can be clinically tested using physical exam techniques such as the Schaeffer test [2, 5]. The Schaeffer test can be performed by asking the patient to flex their wrist and reach their thumb to their pinky finger, forming an "O" shape with their hand. During the maneuver, if present, the palmaris longus muscle should form a bulge anteriorly at the wrist joint [6] as it runs between the flexor carpi radialis tendon medially and the flexor carpi ulnaris tendon laterally.

The palmaris longus muscle is anatomically diverse, with unilateral agenesis being the most frequent of its variants. Other anatomical variants include bifid palmaris longus, triple-headed palmaris longus, accessory palmaris longus, several anomalous insertions at the wrist, and one of the more rare variants, a reversed palmaris longus [7]. In this paper, we present a case of a reversed palmaris longus muscle on the left side in a 55-year-old male cadaver.

## Case presentation

**Figure 1:**
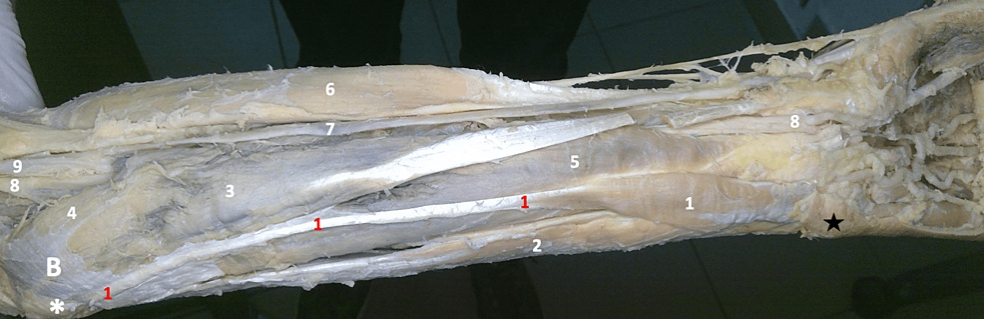
Gross image of the dissected left forearm 1- Reversed Left Palmaris Longus Muscle, 2- Flexor Carpi Ulnaris Muscle, 3- Flexor Carpi Radialis Muscle, 4- Pronator Teres Muscle, 5- Flexor Digitorum Superficialis Muscle, 6- Brachioradialis Muscle, 7- Radial Artery, 8- Median Nerve, 9- Brachial Artery B- Bicipital Aponeurosis (remaining parts of it) ✱ Medial Epicondyle ★ Skin overlying the pisiform

During a regular educational anatomy session (Alfaisal University, College of Medicine, Riyadh, Saudi Arabia), an uncommon and interesting finding of the palmaris longus muscle was uncovered in the cadaver of a 55-year-old male. Throughout the dissection, we followed the Grant’s dissector manual. Digital pictures of the entire dissected area were taken. Of note, all the muscles in the forearm start with the belly proximally and end or taper into a tendon distally. Due to this, the tendon of the reversed muscle was easily discerned, standing out in contrast with and amongst the bellies of the flexors of the wrist and the fingers. Its upper end arises from the area over the common flexor origin and runs distally, immediately medial to the belly of the flexor carpi radialis (FCR) muscle. Tracing it downwards (distally), the tendon was connected to a muscular belly, continuing downwards and medially to then disappear under a triangular tag of skin and fascia at the wrist (Figure 1). 

**Figure 2:**
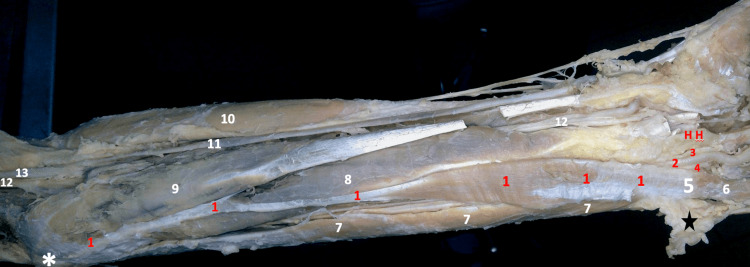
Gross image of the dissected left forearm 1- Reversed Left Palmaris Longus Muscle (the belly of the reversed muscle reaching the pisiform bone), 2- Ulnar Artery, 3- Deep Branch & 4- Superficial Branch of Ulnar Nerve, 5- Pisiform Bone, 6- Abductor Digiti Minimi, 7- Flexor Carpi Ulnaris, 8- Flexor Digitorum Superficialis Muscle, 9- Flexor Carpi Radialis, 10- Brachioradialis Muscle 11- Radial Artery 12- Median Nerve, 13- Brachial Artery HH- Hook of Hamate ✱ Medial epicondyle ★ Skin and fascia are reflected

Considering that the tendon of the reversed muscle is replacing the belly of a normal palmaris longus muscle, which is conventionally known to be the immediate medial structure to the FCR muscle, and since the palmaris longus muscle is renowned for its high tendency of variability, we concluded that the encountered reversed muscle is bound to an aberrant reversed and deviated palmaris longus muscle in the left forearm of that body. The skin tag covering the distal part of the aberrant palmaris longus muscle was reflected, and then meticulous dissection was carried out, separating the muscle from the surrounding fascia to trace the muscle attaching mostly to the pisiform bone; the end of the muscle was partly fused with the surrounding fascia. As the hand was already dissected, the carpal ligaments (palmar and transverse) were absent. The palmar aponeurosis was also resected, rendering it difficult to speculate upon its relation to the distal part of the belly (Figure 2).

**Figure 3:**
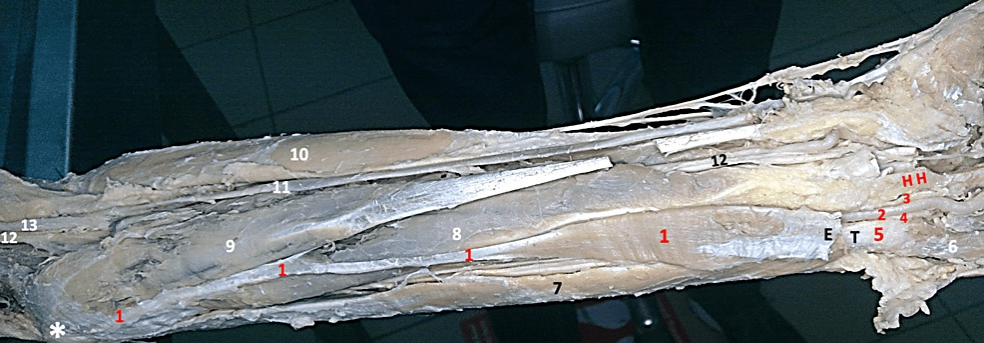
Gross image of the dissected left forearm 1- Reversed Aberrant Left Palmaris Longus Muscle, 2- Ulnar Artery, 3- Superficial Branch of Ulnar Nerve, 4- Deep Branch of Ulnar Nerve, 5- Pisiform Bone, 6- Abductor Digiti Minimi, 7- Flexor Carpi Ulnaris reaching Pisiform, 8- Flexor Digitorum Superficialis Muscle, 9- Flexor Carpi Radialis, 10- Brachioradialis Muscle, 11- Radial Artery, 12- Median Nerve, 13- Brachial Artery ✱ Medial Epicondyle, T- Tendon of Flexor Carpi Ulnaris Muscle reaching Pisiform, E- Cut Edge of the Belly of Palmaris, the removed part of the belly was intimately intermingled with the Palmar Carpal Ligament, reaching the Pisiform Bone , H H- Hook of Hamate N.B. The Transverse Carpal Ligament (flexor retinaculum) and the palmar carpal ligament are removed. 2, 3, and 4 are exposed within the starting point of the Guyon's canal.

Delicate dissection was carried out to expose the ulnar artery and nerve (with its superficial and deep branches) while they were within the Guyon canal (Zone 1). The point of division of the ulnar nerve (into its superficial and deep branches) was retrieved, but was found higher than usual. The radial border of the deviated palmaris longus muscle was 2 cm away from the median nerve at the distal part of the forearm. The distal part of the belly of the muscle was mobilized to trace the superficial and deep branches of the ulnar nerve to their origin point of division. On cutting the distal part of the belly at the point of division of the ulnar nerve, other anatomical structures, like the tendon of the flexor carpi ulnaris muscle reaching the pisiform, were revealed (Figure 3).

**Figure 4:**
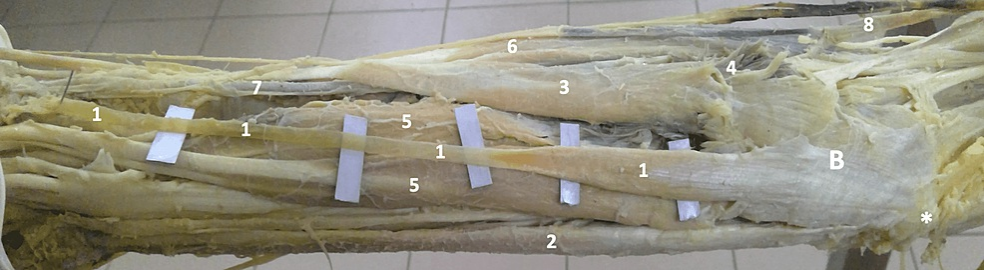
Gross image of the dissected right forearm 1- Normal Right Palmaris Longus Muscle, 2- Flexor Carpi Ulnaris Muscle, 3- Flexor Carpi Radialis Muscle, 4- Pronator Teres Muscle, 5- Flexor Digitorum Superficialis Muscle, 6- Brachioradialis Muscle, 7- Radial Artery, 8- Brachial Artery ✱ Medial Epicondyle B- Bicipital Aponeurosis (remaining parts of it)

This aberrant palmaris longus muscle case presents an anomaly in which the hypertrophy of the medially-deviated reversed belly of the muscle could principally cause compressive ulnar nerve neuropathy, likely involving both of its divisions: the sensory and the motor. On dissection of the contralateral forearm, a normal palmaris longus muscle was revealed (Figure 4).

A video of our cadaver with the palmaris longus muscle on the right side showing a normal orientation, and the palmaris longus on the left side showing a reversed orientation with ulnar deviation, was shown in Video 1. 

**Video 1:** Video Comparison: Orientation of the Palmaris longus in normal vs reversed position with ulnar deviation in our cadaver The video shows the palmaris longus in a normal orientation on the right side and the palmaris longus in a reversed orientation with ulnar deviation on the left side.

## Discussion

In 1973, Still and Kleinert were the first to describe a reversed palmaris longus muscle [8]. A study was conducted in the year 2000, where they described the case of a female patient with a reversed palmaris longus on the right side. In their study, they mentioned all 15 reported cases of reversed palmaris longus prior to that year and that more than 65% of them occurred in females. All 15 cases were on the right side, except for one case that had reversed palmaris longus bilaterally. Furthermore, the median nerve was compressed in 75% of the cases, according to the study [9]. In our case, the reversed palmaris longus muscle was discovered in a male cadaver on the left side of the body, adding to the already uncommon variety.

Carpal tunnel syndrome is caused by entrapment of the median nerve as it passes through the carpal tunnel and makes up 90% of all neuropathies. Pain, paresthesia, and numbness are considered early symptoms of this condition. The symptoms are felt specifically on the first three fingers radially, and on the radial half of the ring finger. As the severity of the compression increases, the patient will start to have additional symptoms, such as clumsiness, weakness in the affected hand, and atrophy of the thenar muscle. Symptoms of the disease most commonly appear at night while lying down and subside during the day in the initial phases. As the illness progresses, symptoms will appear during the day, particularly when performing repetitive tasks like painting, typing, or gaming. Symptoms can become continuous as the condition advances [10]. The tendon of the palmaris muscle is located in close proximity to the transverse carpal ligament. This proximity could raise the carpal tunnel pressure, hypothetically imposing a structural impact on the carpal tunnel, affecting its geometry. As a result, it appears conceivable that people with an intact tendon structure would have a higher incidence of carpal tunnel syndrome [11]. Likewise, the existence of a reversed palmaris longus, in which a bigger muscular part abuts the median nerve, may raise the likelihood of developing carpal tunnel syndrome symptoms significantly.

A study was done to evaluate three female patients who presented with swelling of the anterior region of their wrists and lower forearm. In all three cases, the swelling had impacted their digital flexor tendon, exhibiting carpal tunnel syndrome symptoms. Those patients all reported pain in the lower forearm-to-wrist area, with a minor bluish swelling proximal to the flexor retinaculum, and no obvious thenar wasting. After the tests for rheumatoid arthritis came back negative, the patients then underwent surgery for decompression. The swellings were discovered to be caused by distal bellies of the palmaris longus muscle overlaying a squeezed median nerve located between the belly of the muscle and the supporting tendons [12].

In a study conducted in 2017 on 80 randomly selected and isolated upper limbs, the course and location of tendon insertion, as well as its relationship to the median nerve, were studied. It was found that the palmaris longus muscle can be divided into three major categories based on the morphology of its insertion. The most frequent was type I (78.8%), where the tendon attaches to the palmar aponeurosis. Type II showed a bifurcated tendon, where the lateral division was inserted in the palmar aponeurosis and the medial division in the flexor retinaculum, found in 10 upper limbs (12.5%). The third type was considered the rarest (having only been found in 1.2% of the cases), where the palmaris longus in one limb was found to be fused with the flexor carpi ulnaris muscle. In that group, the insertion was found in the pisiform bone and the palmar aponeurosis in the distal part of the tendon [13]. The third group also includes any insertion morphology that does not fall into type one or two. These categories, however, were made on the premise that the muscle orientation is normal and not reversed. Due to this, the muscle in our case cannot be classified based on its insertion morphology, despite its insertion near the pisiform bone. The muscle here is inverted, and lacks a distal long tendon, giving it a distinctive quality that sets it apart from the three primary groups discovered in the previous study. This inverted nature, along with medial deviation, paired with its insertion near the pisiform bone, contributes to the uniqueness of our case. 

Since the reversed palmaris longus muscle can have an atypical insertion site, it can precipitate inflammation, edema, and blue-colored discoloration, consequently causing pain and numbness in the lower portion of the forearm. This decreases hand functionality, especially when performing hand flexion [14]. Hypertrophy is the ultimate result of repetitive movements of a reversed palmaris longus belly, and this can lead to compartment syndrome, considering the inadequate property of the antebrachial fascia [15].

Guyon canal syndrome is an infrequent peripheral ulnar nerve neuropathy in which the ulnar nerve is injured as it passes through a restricted anatomical pathway at the level of the wrist. The ulnar nerve enters the hand through Guyon's canal, a special spot where the ulnar nerve is susceptible to compression. Inflammation, compression, or trauma, such as hook of hamate fractures, can all result in distal ulnar nerve injury. Other causes include lipoma and excess fat tissue within the Guyon canal. In rare cases, an aberrant muscle can lead to ulnar nerve damage [16]. The relationship between having a reversed palmaris longus and ulnar neuropathy has been previously reported in the literature [7, 17, 18].

In the field of hand reconstruction surgery, the palmaris longus tendon is frequently used as it is thought to be the best source for grafting the hand's long flexors [19]. Due to its close proximity to vital nerves and arteries, it is critical to understand the potential variation in the palmaris longus muscle, as this variation may influence the choice of the palmaris longus tendon in grafting [13].

## Conclusions

It is not uncommon to have different morphological variations of the palmaris longus muscle. These variations include different insertion sites, an absent, bifurcated, hypertrophied, and reversed muscle. Our case here discussed one of the rare variants, a unilaterally reversed palmaris longus muscle in the left forearm, and its clinical significance in the literature. In a reversed case, the orientation and position of the muscle can affect or compress the nerves surrounding it, contributing to various pathologies such as carpal tunnel syndrome and Guyon's canal syndrome, especially when the muscle hypertrophies with repetitive use. Despite having been deemed inert, the palmaris longus muscle has always been an important surgical landmark. Its variations play a critical role in surgical settings, as it is considered one of the best sources of grafts, especially in reconstructive surgeries. Carefully studying the variations in this muscle will also save the surgeons any unnecessary surgical complications. 
